# An Efficient Technique for Ammonia Capture in the Haber–Bosch Process Loop—Membrane-Assisted Gas Absorption

**DOI:** 10.3390/polym14112214

**Published:** 2022-05-30

**Authors:** Anton N. Petukhov, Artem A. Atlaskin, Kirill A. Smorodin, Sergey S. Kryuchkov, Dmitriy M. Zarubin, Maria E. Atlaskina, Anastasia N. Petukhova, Anna N. Stepakova, Anna A. Golovacheva, Artem N. Markov, Ekaterina A. Stepanova, Andrey V. Vorotyntsev, Ilya V. Vorotyntsev

**Affiliations:** 1 Nanotechnology and Biotechnology Department, Nizhny Novgorod State Technical University n.a. R.E. Alekseev, Minin St. 24, 603950 Nizhny Novgorod, Russia; antopetukhov@gmail.com; 2 Laboratory of Smart Materials and Technologies, Mendeleev University of Chemical Technology of Russia, Miusskaya Sq. 9, 125047 Moscow, Russia; kriuchkov.ss@gmail.com (S.S.K.); atlaskina.m.e@gmail.com (M.E.A.); anassini@gmail.com (A.N.P.); stepakova.a.n@muctr.ru (A.N.S.); ilyavorotyntsev@gmail.com (I.V.V.); 3 Chemical Engineering Laboratory, N.I. Lobachevsky State University of Nizhny Novgorod, 603022 Nizhny Novgorod, Russia; smorodin.kirill.a@gmail.com (K.A.S.); dimazarubin493@gmail.com (D.M.Z.); a.golovacheva.chem@yandex.ru (A.A.G.); markov.art.nik@gmail.com (A.N.M.); stepanova.k1999@mail.ru (E.A.S.); an.vorotyntsev@gmail.com (A.V.V.)

**Keywords:** membrane-assisted gas absorption, ammonia recovery, separation efficiency, deep eutectic solvents, hollow fibers

## Abstract

The present study continues the development and enhancement of a highly efficient unique hybrid technique—membrane-assisted gas absorption in designing the separation unit, which provides the improvement in mass-transfer of a target component during the ammonia capture process from a process loop of the Haber–Bosch technological route. In order to minimize the absorbent volume to membrane area ratio, the special separation cell was designed based on a combination of two types of hollow fiber membranes, dense gas separation membrane and porous pervaporation membrane. The separation performance tests were implemented under two sets of conditions, sweeping the bore (permeate) side of a cell with helium and hydrogen-nitrogen mix. For both cases, the membrane-assisted gas absorption cell demonstrated high separation efficiency, and the ammonia concentration in the permeate was never lower than 81 mol%; meanwhile, under the hydrogen-nitrogen bore sweep conditions, the ammonia concentration in the permeate reached 97.5 mol% in a single-step process. Nevertheless, there is a product purity–recovery rate trade-off, which is a typical issue for separation processes.

## 1. Introduction

At present, global ammonia and nitrogen fertilizers production has a leading position among the global chemical industry. The annual capacity of ammonia produced worldwide in 2021 was equal to 236.4 million metric tons, and projected growth by 2030 is about 290 million metric tons [1]. Most of the produced ammonia was synthesized using the Haber–Bosch process invented in 1909 [2], which became conventional over the 20th century and allowed the expansion of ammonia synthesis worldwide from about 3–4 million metric tons per year (1945) to present capacity.

Despite the simplicity of the straightforward reaction, sustainable process, and stable product, the key technological steps in the route have a number of drawbacks [3,4]. For instance, the ammonia recovery unit based on condensation is incapable of capturing all produced ammonia. Therefore, a significant amount of uncaptured ammonia is recycled to the reactor, where the chemical equilibrium shifts towards reagents and decreases the unit performance.

Another critical issue in the Haber–Bosch process is the source of hydrogen conventionally produced by hydrocarbons reforming, which does not fit the present zero-emission strategy due to the generation of considerable carbon monoxide and dioxide exhaust. The so-called “green” ammonia process [5,6] seems to be a prospective solution, where the hydrogen is produced via pure water dialysis and the nitrogen is produced from air using membranes. Both unit gears are powered by electricity generated with renewable sources such as windmills and/or solar cells.

In this way, it is evident that process optimization should be performed, taking into account factors listed above to enhance both the performance of the plant and the product recovery. As the ammonia capture stage does not provide sufficient product capture, a significant amount of ammonia is recycled to the reactor, where the chemical equilibrium shifts towards the reagents and decreases the product yield. Capturing ammonia from the recycling loop will enhance the product recovery and the chemical equilibrium in the reactor will shift towards the product, resulting in a productivity increase. Meanwhile, the same amount of inlet hydrogen and nitrogen being converted into a surplus amount of ammonia or less source gas will maintain production power.

Membrane-assisted gas absorption unit with built-in technological scheme provides selective mass transfer between recycled and feed streams that allows capture of the residual ammonia and sends it back to the refrigeration unit. Previously, the invented membrane-assisted gas absorption unit was studied comprehensively in the context of ammonia capture from binary and ternary gas mixtures and in the acid gases removal applications [7,8,9,10,11]. Moreover, recently, the lab-scale unit based on the flat membrane was tested concerning the most efficient adsorbent in a so-called “closed-mode operation” with a zero retentate [7]. The obtained separation results proved the efficiency of the proposed technique. Thus, studying the separation efficiency during the separation of a ternary gas mixture consisting of NH_3_/H_2_/N_2_ in a volume ratio of 15.5/62.8/21.7 vol%, it was observed that it is possible to produce an ammonia stream on the permeate side with purity of 98.7 vol.%.

The membrane-assisted gas absorption technique is a hybrid pressure-driven process, where the separation occurs in continuous mode through the absorption of gases by liquid absorbent covering the membrane with further permeation through it. In this way, the separation is running in the absence of phase transitions, at ambient temperature, and in a single stage. The appliance of a liquid absorbent provides enhanced selectivity compared to the conventional membrane gas separation process. The key feature of that technique is the spontaneous absorbent regeneration, which also occurs in the continuous mode as gas desorbs and passes through the membrane beneath. As a consequence, despite all the engineering features, the absorbent and membrane materials are the key. Because of that, the absorbent and membrane should be chosen wisely, taking into account mass transfer rates.

At present, special attention is given to the deep eutectic solvents (DESs) [12,13,14], which seem to be a prospective alternative to recently widely studied room temperature ionic liquids (RTILs) [15,16,17], which have proven to be materials with high absorption capacity with regard to ammonia. Deep eutectic solvents possess both similar loading as RTILs and the merits of low cost. In addition, RTILs’ deep eutectic solvents are characterized by renewability and low toxicity. Unlike the RTILs, DESs are more beneficial for chemical industry as their production is simple and performed through mixing in the suitable molar ratio of hydrogen-bond donors and acceptors.

A number of ammonium thiocyanate-based DESs were studied on the example of ammonia capture using the membrane-assisted gas absorption as well [12]. Among the three promising candidates (ammonium thiocyanate: glycerol 2:3, ammonium thiocyanate: ethylene glycol 1:3, and ammonium thiocyanate: urea 2:3) studied comprehensively by Deng et al., ammonium thiocyanate: glycerol provides the highest ammonia loading (176.4 g of NH_3_ per kg of DES); meanwhile, it is the most viscous DES in the list. Ammonium thiocyanate: ethylene glycol provides comparable ammonia loading of 168.4 g of NH_3_ per kg of DES and six-times lower viscosity, which is more preferable for mass transfer applications due to the higher diffusion rate of the gases. Nevertheless, the complex material screening is of great importance to enhance the membrane-assisted gas absorption performance by wise and accurate combination of membrane and absorbent.

This research study deals with a highly efficient unique hybrid technique—membrane-assisted gas absorption in designing the separation unit. In order to enhance the separation efficiency and to lower the absorbent volume to membrane area ratio, the special cell was designed based on a combination of two different types of hollow fiber membranes with a dense and porous selective layer. The designed cell separation performance was tested under two different conditions, namely sweeping the permeate side with helium (ideal conditions) and with hydrogen-nitrogen mix (quasi real conditions), and the ammonia capture efficiency was evaluated, taking into account its concentration in the permeate and retentate flows.

## 2. Materials and Methods

### 2.1. Materials

According to the purpose of the current study—to evaluate the efficiency of a novel membrane-assisted gas separation unit in the ammonia recovery step of the synthesis technological route—the specific gas mixtures were prepared and sealed in the stainless-steel cylinders. The first one, which contains ammonia to be captured, is identical to the stream leaving the refrigeration block and is recycled to the reactor. It mainly consists of hydrogen and nitrogen, with a small portion of methane, ammonia, and argon, and the proportion is as follows: H_2_/N_2_/CH_4_/NH_3_/Ar = 62.53/23.1/7.49/2.38/4.5 mol%. The second mixture contains 75 mol% of hydrogen and 25 mol% of nitrogen. That gas mixture is almost equal in composition to the gas stream moving toward the refrigeration block except for the low content of methane and argon (1.03 and 0.27 mol%, respectively). The preparation of gas mixtures was performed using single gases of high purity: NH_3_ (≥99.9999 vol.%) was purchased from Firm HORST Ltd. (Dzerzhinsk, Russia); H_2_ (≥99.9999 vol.%), N_2_ (≥99.99 vol.%), CH_4_ (≥99.995 vol.%), and Ar (≥99.9999 vol.%) were purchased from NIIKM Ltd. (Moscow, Russia).

The deep eutectic solvent was used as liquid absorbent in the ammonia capturing process using a membrane-assisted gas absorption unit. All reagents needed for the preparations of DES were purchased from Sigma Aldrich Group (Taufkirchen, Germany). In the preparation of specific deep eutectic solvent, the following hydrogen-bond acceptor and donor were used: ammonium thiocyanate (≥99.99%) and urea (≥99.5%). No additional purification of reagents was used and the standard gravimetric method was used. No additional measurements devoted to the moisture content in the prepared DES were performed, as it was prepared under the dry nitrogen environment, further it was sealed in the flask and then was placed in the membrane-assisted gas separation cell connecting the flask to one container and the vacuum pump connected to another container. In this way, there was no contact of prepared DES with the room atmosphere. Taking into account the mixed gas preparation method, there is no source of moisture in the experimental setup, so the influence of water on separation efficiency was not conducted

### 2.2. Absorbents Screening

The data, obtained during the comprehensive screening of a wide range of absorbent materials is given in Table 1.

In ref. [18], the authors determined the solubility of ammonia in deep eutectic solvents consisting of choline chloride (ChCl) and urea taken in three different molar ratios (2:3, 1:2, and 2:5). The experiment was carried out in the temperature range 298.2–353.2 K and pressure from 0 to 300 kPa. The results showed that the solubility of NH_3_ in ChCl+urea (1:2) was somewhat higher than in the other two DESs tested. This is explained by the lowest melting point among the liquids studied in the work. According to the authors, this indicates a less dense aggregation of molecules, providing more free volume for gas dissolution.

A class of highly effective deep eutectic solvents for the isolation of NH_3_ was developed, synthesized, and studied in [19]. Synthesized DESs are composed of choline chloride (ChCl), phenol (PhOH), and ethylene glycol (EG). By exploiting the weak acidity of PhOH, a highly efficient and reversible absorption of NH_3_ was realized in PhOH-based triple DESs. The solubility of NH_3_ in prepared DESs can reach 9.619 mol/kg (0.164 g/g) at 298.2 K and 101.3 kPa, which is one of the best values to date.

A study of deep eutectic solvents based on choline chloride using glycerol, ethylene glycol, N-methyl urea, and trifluoroacetamide as HBDs with a fixed molar ratio of 1:2 [20] showed that DES containing glycerol (0.051 g/g) has the highest NH_3_ solubility.

In ref. [21], a study was made of deep eutectic solvents based on choline chloride (ChCl) with the addition of dihydric alcohols: 1,4-butanediol (1,4-BD), 2,3-butanediol (2,3-BD), and 1, 3-propanediol (1,3-PD) in various molar ratios in the temperature range of 303.15–333.15 K and pressures from 25 to 420 kPa. The highest solubility of NH_3_ (0.06788 g/g) at 132.8 kPa and 303.15 K was shown by DES ChCl + 1,3-PD (1:4).

Choline chloride DESs using weakly acid azoles, in particular, imidazole (ImZ), triazole (TrZ), and tetrazole (TetrZ) in combination with ethylene glycol (EG) were considered in [22]. DES ChCl + TetrZ + EG showed the highest sorption capacity for ammonia. At a pressure of 104 kPa and a temperature of 313.2 K, the solubility of ammonia was 0.16948 g/g.

In ref. [23], the sorption capacity of ammonia was studied in a number of DESs: choline chloride resorcinol (ChCl + Res), choline chloride phenol (ChCl + Phe), choline chloride glycerol (ChCl + Gly), choline chloride phenol ethylene glycol (ChCl + Phe + EG), choline chloride resorcinol glycerin (ChCl + Res + Gly), choline chloride fructose glycerin (ChCl + D-fructose + Gly), choline chloride malic acid glycerin (ChCl + DL-malic Acid + Gly), choline chloride levulinic acid glycerin (ChCl + Levulinic Acid + Gly), choline chloride oxalic acid glycerin (ChCl + Oxalic Acid + Gly), choline chloride malonic acid glycerin (ChCl + Malonic Acid + Gly), and choline chloride phenylacetic acid glycerol (ChCl + Phenylacetic Acid + Gly). Among the considered liquids, DES ChCl + Res + Gly had the highest ammonia solubility. At a temperature of 313.15 K and a pressure of 101.3 kPa, the sorption capacity was 0.13 g/g.

Deep eutectic solvents based on ethylamine hydrochloride (EaCl) and urea (Urea) were tested in 3 different ratios: 1:2; 1:1; 2:1 in [24]. The most effective was the DES with a molar ratio of EaCl to Urea 1:1 at a temperature of 313.2 K and a pressure close to atmospheric. The ammonia capacity of the absorbent was 0.07788 g/g.

The values of NH_3_ absorption in prepared mixtures of EaCl+Gly were experimentally measured [25] at various temperatures and pressures. The NH_3_ capacities of EaCl+Gly mixtures were found to be quite impressive, with a maximum value of 9.631 mol/kg at 298.2 K and 106.7 kPa.

DES, composed of protic ethanolamine hydrochloride (EaCl), weakly acidic resorcinol (Res), and neutral glycerol (Gly), is capable of absorbing 0.240 g NH_3_/g DES for EaCl/Res/Gly (1:4:5) at 293 K and 0. 1 MPa, which is much higher than most registered DESs. In addition, as reported by the authors in [26], DES EaCl/Res/Gly (1:4:5) has good reversibility, excellent NH_3_/CO_2_ selectivity, and is a potential NH_3_ absorption and storage material for industrial applications.

The ref. [27] describes the sorption capacity of DESs of ethylamine hydrochlorides in combination with phenol in various ratios. The results of the study showed that the solution with the highest proportion of phenol-EaCl+PhOH (1:7), −0.167 g/g at a temperature of 298.2 K and atmospheric pressure, has the highest ammonia solubility.

Ammonium thiocyanate (NH_4_SCN) was used in [12] as an acceptor of hydrogen bonds in DES for ammonia sorption. Glycerol (Gly), ethylene glycol (EG), urea (Urea), acetamide (AT), and caprolactam (CL) were taken as hydrogen bond donors. The results of the experiment showed a high sorption capacity of sorbents with glycerol, ethylene glycol, and urea. The highest ammonia capacity was observed in DES NH_4_SCN + Gly and amounted to 0.17642 g/g. Such indicators of sorption capacity turned out to be one of the highest in comparison with the other sorbents considered in the report, which indicates a good prospect for the use of DESs based on ammonium thiocyanate in the tasks of ammonia recovery.

In the ref. [28], NH_4_SCN was paired with imidazole (Im) DESs to form low-viscous DESs with dual active sites for highly efficient NH_3_ absorption. The effects of absorption temperature, gas flow rate, water content, and molar ratio on NH_3_ absorption performance as well as physical properties of NH_4_SCN/Im DESs were systematically investigated. The absorption capacity of NH_4_SCN/Im (1:2) DES reached 9.65 mol NH_3_/kg DES for atmospheric NH_3_.

In a ref. [29], the authors found that N-methylacetamide (MAA) can form DES with heterocyclic weak acids (HWA) such as imidazole, 1,2,4-triazole, and tetrazole (TetrZ). DES MAA + tetrazole are among the best solvents for NH_3_ absorption with NH_3_ solubility of 0.13624 g/g at 313.2 K and 102.9 kPa.

Glycerol (Gly) as a hydrogen bond donor and 1,2,4-triazole (Tri), imidazole (Im), and tetrazole (Tz) as hydrogen bond acceptors were taken for the synthesis of DESs to study the sorption capacity of ammonia [30]. DES containing tetrazole showed the best result, with an ammonia sorption capacity of 0.179 g/g.

Akhmetshina et al. [31] studied the sorption capacity of DES, in which the ionic liquid 1-butyl-3-methylimidazolium methanesulfonate was the hydrogen bond acceptor, and urea was the hydrogen bond donor. The sorption capacity of the obtained DES turned out to be below the average level and amounted to 0.01787 g/g at 172.6 kPa and 313.2 K.

An effective strategy was proposed by combining the protic ILs (PILs) with acidic H and low viscous ethyleneglycol (EG) to form IL-based deep eutectic solvents (DESs) for NH_3_ absorption [32]. The highest mass capacity of 211 mg NH_3_/g DES was achieved by [Im][NO_3_]/EG with molar ratio of 1:3.

In ref. [33], imidazole/resorcinol DESs were studied in different ratios of components (1:1, 1.5:1, 2:1, 2.5:1). The results displayed that binary DES imidazole/resorcinol (1:1) can absorb up to 0.238 g NH_3_ per g DES at 293.15 K and 0.1 MPa.

Two deep eutectic solvents consisting of urea (U) and choline salts such as dimethyl-di(2-hydroxyethyl)-ammonium chloride [Me_2_COH_2_N]Cl and methyl-tri(2-hydroxyethyl)-ammonium chloride [MeCOH_3_N]Cl prepared in a ratio of 1:1 have been extensively researched for ammonia absorption applications. Kazarina O.V. and co-authors [13] carefully studied their densities, viscosities, refractive indices, and properties associated with the absorption of ammonia; it was found that the resulting DESs have an absorption capacity of 2.078 and 2.632 mol NH_3_ kg^−1^ DES for [Me_2_COH_2_N]Cl/U and [MeCOH_3_N]Cl/U, respectively, at 313.2 K and 101.3 kPa, which is about two times higher than for choline chloride/urea (2:3) DES under the same conditions.

A study of the sorption capacity of NH_3_ in protic ionic liquids showed that the length of the cation chain has little effect on the solubility of NH_3_ [34]. For this, three ionic liquids were taken: 1-methylimidazolium bis(trifluoromethylsulfonyl)imide ([mim][Tf_2_N]), 1-ethylimidazolium bis(trifluoromethylsulfonyl)imide ([eim][Tf_2_N]), 1,2-dimethylimidazolium bis(trifluoromethylsulfonyl)imide ([mmim][Tf_2_N]), the sorption capacity of ammonia in which practically does not change. The solubility of NH_3_ in ionic liquids was also evaluated in the work: 1-butylimidazolium bis(trifluoromethylsulfonyl)imide ([bim][Tf_2_N]), 1-butylimidazolium thiocyanide ([Bim][SCN]), 1-butylimidazolium nitrate ([Bim ][NO_3_]), 1-butyl-3-methylimidazolium thiocyanide ([Bmim][SCN]), 1-butyl-3-methylimidazolium dicyandiamide ([Bmim][DCA]), 1-butyl-2,3-dimethylimidazolium bis (trifluoromethylsulfonyl)imide ([Bmmim][Tf_2_N]), 1-butyl-2,3-dimethylimidazolium thiocyanide ([Bmmim][SCN]), 1-butyl-2,3-dimethylimidazolium dicyandiamide ([Bmmim][DCA]), 1-butyl-3-methylimidazolium bis(trifluoromethylsulfonyl)imide ([Bmim][Tf_2_N]). Taking into account the effect of anions with the same [Bim]+ cation, the order of solubility of NH_3_ in proton ILs was [Bim][Tf_2_N]> [Bim][SCN]> [Bim][NO_3_].

A. Yokozeki and M.B. Schiflert conducted a study in which they experimentally evaluated the solubility of ammonia in a number of ionic liquids: 1-butyl-3-methylimidazolium hexafluorophosphate ([bmim][PF_6_]), 1-hexyl-3-methylimidazolium chloride ([hmim][Cl]), 1-ethyl-3-methylimidazolium bis(trifluoromethylsulfonyl)imide ([emim][Tf_2_N]), 1-butyl-3-methylimidazolium bis(trifluoromethylsulfonyl)imide ([bmim][Tf_2_N]), and 1-butyl-3- methylimidazolium tetrafluoroborate ([bmim][BF_4_]) [35]. The IL [bmim][PF_6_] had the highest sorption capacity for ammonia (0.02101 g/g) at 298.2 K and 101.3 kPa.

Li, Zhou and other authors of the study [36] found an increase in the sorption capacity for ammonia in ionic liquids with an increase in the length of the alkyl cations. The work was carried out using ionic liquids with one anion—tetrafluoroborate ([BF4]^−^) and four different cations: 1-ethyl-3-methylimidazolium ([emim]^+^), 1-butyl-3-methylimidazolium ([bmim]^+^), 1-hexyl-3-methylimidazolium ([hmim]^+^), and 1-octyl-3-methylimidazolium ([omim]^+^). It was found that the solubility of ammonia increases in the series [omim][BF_4_] > [hmim][BF_4_] > [bmim][BF_4_] > [emim][BF_4_]. Thus, the IL [omim][BF_4_] had the highest ammonia sorption capacity (0.2333 g/g) at 120 kPa and 298.15 K.

A class of ionic liquids based on 1,1,3,3-tetramethylguanidinium (TMG) with anions: tetrafluoroborate ([BF_4_]^−^), bis(trifluoromethylsulfonyl)imide ([Tf_2_N]-) was synthesized and studied in [37]. It was found that the absorbent [TMGH][BF_4_] had the highest ammonia solubility (0.09006 g/g) at 101.3 kPa and 293.2 K.

In ref. [38], the following was studied: a series of hydroxyl-functionalized 1-2(-hydrohexyl)-3-methylimidazolium ILs ([EtOHmim]) with anions of thiocyanide (SCN), nitrate (NO_3_), hexafluorophosphate (PF_6_), tetrafluoroborate (BF_4_), bis(trifluoromethylsulfonyl)imide (Tf_2_N), and dicyandiamide (DCA). Among the considered ILs, the [EtOHmim][BF_4_] IL had the highest ammonia solubility, being able to absorb 0.045 g of NH_3_ per gram of IL at 101.3 kPa and 313.15 K.

The following was studied: the solubilities of ammonia in room temperature ionic liquids 1-ethyl-3-methylimidazolium acetate [emim][Ac], 1-ethyl-3-methylimidazolium thiocyanate [emim][SCN], 1-ethyl-3-methylimidazolium ethylsulfate [emim][EtOSO_3_], and N,N-dimethylethanolammonium acetate [DMEA][Ac]. Among the considered ILs, [DMEA][Ac] had the highest solubility, the capacity of which is 0.10006 g/g at 298.1 K and 101.3 kPa [39].

In ref. [40], the experimental values of densities, viscosities, and refractive indices at 298.15 K and atmospheric pressure are reported for ternary and coupled binary mixtures of tris(2-hydroxyethyl)methylammonium methylsulfate [MTEOA][MeOSO_3_]. The solubility of ammonia in the studied IL was 0.082 g/g.

Kazarina O.V. and other authors of the study [17] studied ammonia sorption in novel room temperature ionic liquids (RTILs) such as dimethyl (IL-4) and methyl mono-(di-)(2-hydroxyethyl) (2-hydroxyethoxy)ethyl chloride (IL- 5), all of which were characterized by FT-IR, 1H, and 13C NMR.

The paper [41] explores a number of multidimensional covalent organic frameworks (COFs) that are densely functionalized with various active groups such as –N–H, –C=O, and a carboxyl group. Due to the synergistic multidimensional and exposed metal site, COF materials show excellent adsorption capacity (0.15915 g/g for [HOOC]_17_-COFs at 100 kPa and 298 K).

A study of the solubility of NH_3_ in the ionic liquid 1-n-butyrate-3-methylimidazolium bis(trifluoromethylsulfonyl)imide ([HOOC(CH_2_)_3_mim][NTf_2_]) showed the following result: 0.058 g of ammonia dissolves in 1 g of IL [42]. At a temperature of 293.15 K, the sorbent is characterized by high viscosity—more than 1500 mPa s, which sharply decreases upon heating, and at a temperature of 313.15 K, it is about 350 mPa s.

### 2.3. Experimental Setup

**Figure 1:**
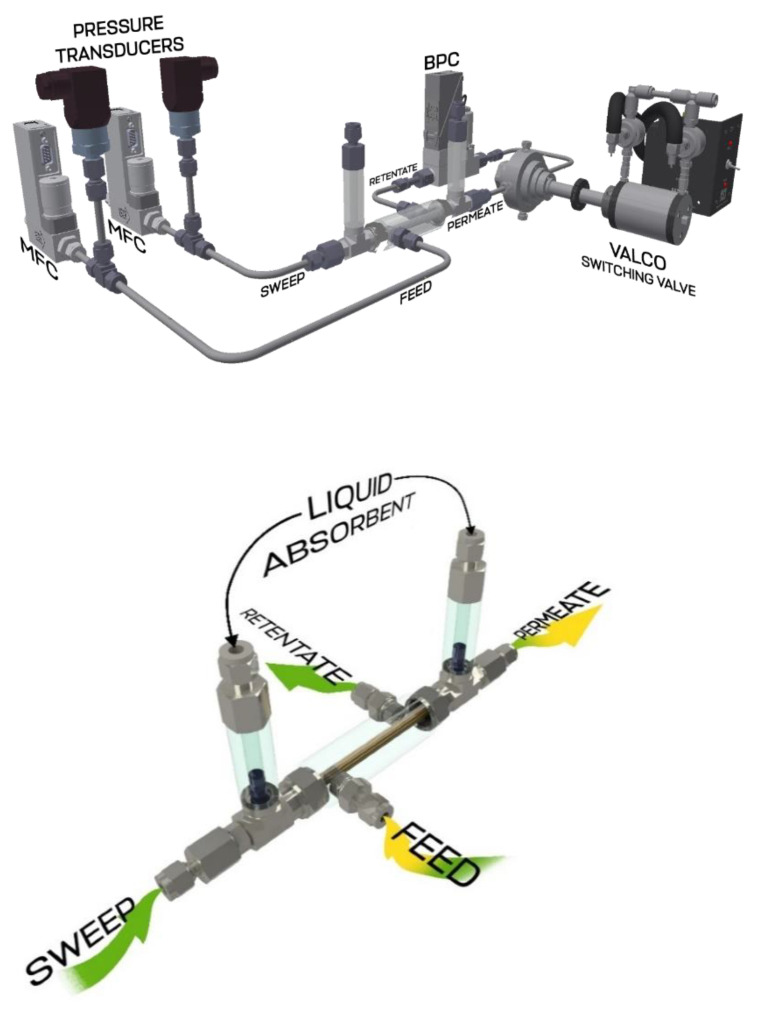

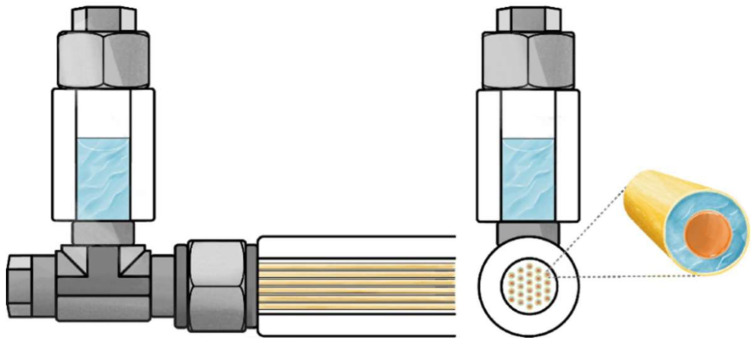
The 3D scheme of experimental setup for membrane-assisted gas absorption cell separation performance test (on the top), 3D image of the membrane-assisted gas separation cell (in the middle), and its principal scheme (bottom).

The principal scheme of the experimental setup on the top part, the 3D image of the membrane-assisted gas separation cell in the middle, and its scheme on the bottom are shown in Figure 1. In that unit, the separation occurs under the counter-current flow mode. In this way, the feed and permeate streams are connected to one side of the separation cell; meanwhile, the retentate and permeate sweep are on the opposite of the cell. Both the feed and the permeate sweep stream are equipped with high-precision mass flow controller provided by Bronkhorst (El-Flow Prestige FG-201CV, Veenendaal, Netherlands) and pressure transmitters by Wika (S-20), Klingenberg, Germany. In order to maintain constant pressure during the separation process, the retentate line is equipped with a back pressure controller (EL-Press P-702CM) by Bronkhorst, Veenendaal, Netherlands. The permeate line pressure is a process-controlled value and is formed by the pneumatic resistance in the fibers and permeated gas stream; in other words, it is not maintained manually by the operator. The process outlet streams, retentate and permeate, are connected to the pneumatic actuated selector valve by VICI Valco (A4VL4MWE2, Schenkon, Switzerland), equipped with a high-speed switching accessory (HSSA), which allows performing quick switching, requiring only 8 ms. It is used to perform alternate switching of the streams to be analyzed with the gas chromatography system and this device does not create pneumatic resistance in the line, which is typical for the common valves, where the switching time is more than 180 ms. After the stream of interest is determined, it flows toward the analytical system presented gas chromatograph by Chromos, where the sample is separated in column under isothermal conditions and is detected by the thermal conductivity detector (TCD). The detailed GC-analysis conditions are presented in Table 2.

The experimental procedure includes the supply of gas mixture to the feed inlet of the experimental setup through the pressure regulator DRASTAR, maintaining the constant pressure before the mass flow controller which provides the accurate flow rate of a mixture to be separated. Feed enters the membrane-assisted gas separation cell, where ammonia is captured using a combined membrane-absorbent system and moved to the permeate stream. Further, the permeated ammonia stream is picked up with a sweep supplied through the pressure and mass flow controllers and moves out of the cell as well as the retentate stream, which is now depleted of ammonia. The back pressure controller mounted on the retentate line maintains the constant value along the whole line from feed to itself and guarantees constant pressure difference across the combined system. Both the permeate and retentate stream samples are alternately analyzed using the GC system [7] to obtain the dynamics of reaching the steady-state and separation performance. Experimental conditions in detail are given in Table 3.

### 2.4. Membrane-Assisted Gas Absorption Cell Design

**Figure 2:**
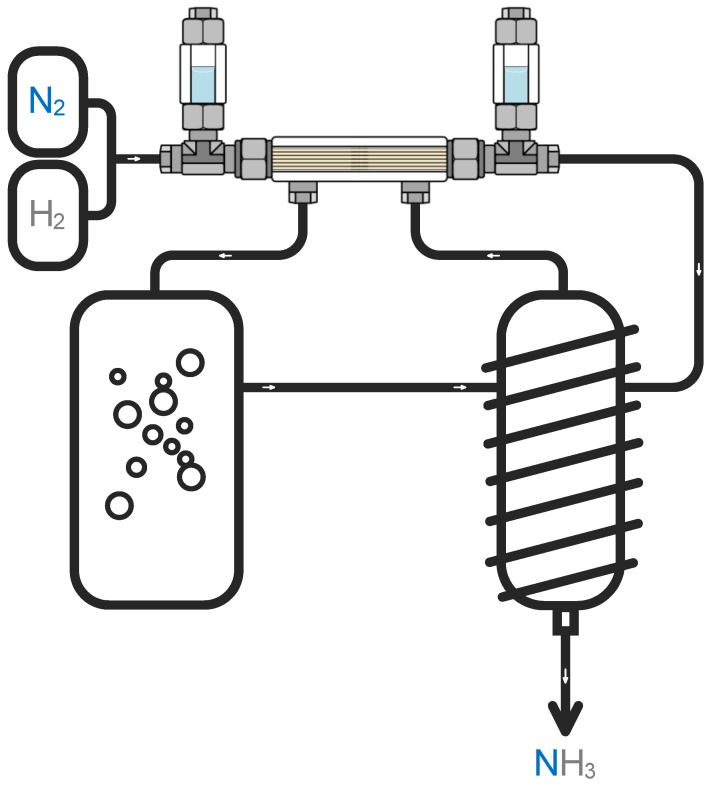
The principal scheme of the Haber–Bosch process with built-in membrane-assisted gas absorption unit.

Here, the special separation cell design was proposed to implement capture of the NH_3_ from the recycle loop of the Haber–Bosch process (Figure 2). Here, two different types of hollow fiber membranes are used: pervaporation hollow fiber membrane PS-50, provided by the Laboratory of Membrane Processes of the Institute of Physical Organic Chemistry of the National Academy of Sciences of Belarus and gas separation asymmetric hollow fiber membrane made of polysulfone provided by Hangzhou Kelin Aier Qiyuan Equipment Co., Ltd. (Hangzhou, China) Plexiglas made of polymethyl methacrylate was used as a shell of the membrane module in order to be able to visually monitor the process.

The key feature of the membrane-assisted gas separation cell is the implementation of a combined system of two types of hollow fiber polymer membranes. In the end parts of the module housing, a special sealing compound is used to fix the pervaporation polymer fiber used to provide the contact between two phases (the gas mixture to be separated and absorbing liquid). The gas separation hollow fiber membrane is placed inside of the pervaporation fiber and is used to remove the continuously desorbed gas phase from the liquid. The fixation and sealing of the gas separation hollow fibers are made not in the end parts of the module shell, as in the case of pervaporation fiber, but along the edges of the pipe fittings—tees, fixed on the ends of the module shell. This configuration allows the formation of a gap between the two types of fibers and eliminates the possibility of liquid entering the gas separation hollow fibers. The liquid absorbent is loaded into special cylindrical containers placed on a tee connecting the shell of the separation cell with the gas distribution system. The containers with the absorbent are mounted between the ends of the hollow fibers so the liquid is in the gap formed by the two fibers. Thus, the liquid absorbent is located in a closed volume between two hollow polymer fibers, through one of which the feed gas mixture contacts the liquid absorbent, and through the other the absorbed component is removed. On the outer surface of the cylindrical shell of the separation cell, two nipples are fixed: one is the feed mixture inlet and the other is the retentate outlet.

The separation process occurs as follows. One of the nipples fixed on the outer surface of the separation cell shell is supplied with a feed mixture flow, which fills the internal volume of the cell and contacts the liquid absorbent, located in the gap between two fibers, through the pervaporation fibers. Further, the components, which are dissolved in the absorbent, are removed from it under the pressure gradient and move to the permeate side of the cell through the gas separation hollow fiber and form a stream enriched with a high-soluble component. Components that are characterized by low solubility in the used absorbent form a retentate stream and are removed from the membrane-assisted separation cell through a nipple mounted on the opposite side of the shell. The permeate side of the designed module is a flow-through volume and allows mass transfer between two recirculation circuits of gas mixtures in the process of ammonia synthesis.

### 2.5. Membrane Permeance Test

**Figure 3:**
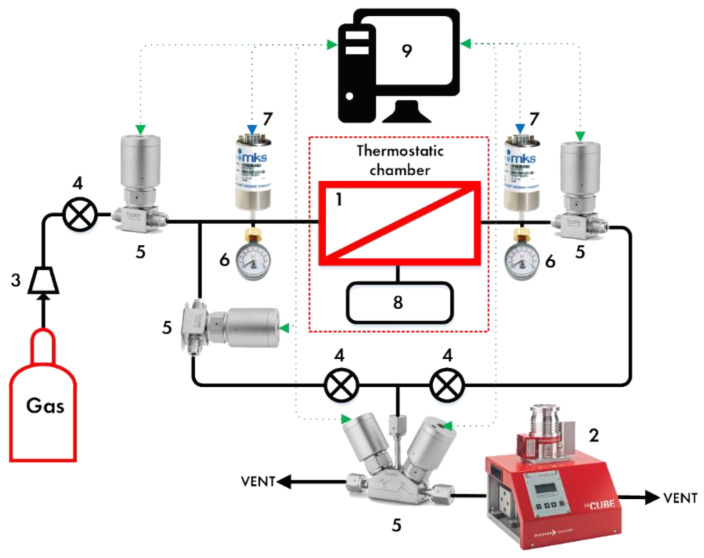
The principal scheme of time-lag setup. 1—membrane test cell; 2—vacuum station; 3—manual pressure regulator; 4—diaphragm manual valves; 5—diaphragm valves with pneumatic actuators; 6—pressure gauges; 7—pressure transducers; 8—additional volume; 9—personal computer.

The ideal gas transport characteristics of hollow fibers were determined using a well-known time-lag experimental setup (Dynes–Barrer method) [43,44]. The setup principal scheme is given in Figure 3. The gas distribution system includes a membrane test cell (1), a vacuum station (2), (Pfeiffer Hi-Cube 80 ECO) manual pressure regulator (3), RPA1 (A-Flow), diaphragm manual valves (4), SS-DSS4 (Swagelok) diaphragm valves with pneumatic actuators (5), 6LVV-DPFR4-P1-C (Swagelok) pressure transducers (6): 0–16 bar (Wika S-10) on the feed side and 0–100 Torr on the permeate side (MKS Instruments 750B), pressure gauges (7): −0.1–1.5 MPa on the feed side and −0.1–0.3 MPa on the permeate side.

Once the membrane sample was placed in the test cell, the entire system was evacuated to a residual pressure of <0.13 kPa. After that, the system was disconnected from the vacuum pump and pure gases (N_2_, H_2_, CH_4_, Ar, and NH_3_) were sequentially supplied to it under a pressure of 101.325 kPa. Between each test, the system was purged with helium and evacuated using a vacuum station during the 2 h.

Permeance *Q* was calculated according to:

(1) $$Q=\frac{{Vp}_{2}}{V_{m}p_{0}}\frac{1}{S\tau \left(p_{1}-p_{2}\right)}$$

where *V*—permeate side volume (mL); *V_m_*—molar volume (mL/mol); *p*_0_—atmospheric pressure (Pa); *p*_1_—pressure on the feed side (Pa); *S*—effective membrane area (m^2^); *τ*—duration of the experiment (s); *p*_2_—pressure on the permeate side (Pa). Next, the permeance was converted into GPU units, using the following equation:

(2) $$1\;\mathrm{GPU}=3.346\times 10^{-10}\frac{\mathrm{mol}\;}{m^{2}\;s\;\mathrm{Pa}}$$

Ideal selectivity of the sample is calculated from:

(3) $$\alpha =Q_{A}/Q_{B}$$

where *Q_A_* and *Q_B_* are the permeance of gases *A* and *B*.

## 3. Results and Discussion

### 3.1. Membrane Permeance

After the appropriate liquid absorbent was chosen based on comprehensive material screening (Section 2.2), the critical issue is the suitable membrane material, taking into account its mass transfer properties (permeance, selectivity) and stability in the presence of plasticizing components, such as ammonia. Recently, Petukhov A.N. et al. presented their study, containing their conclusions on the most suitable absorbents and membranes for membrane-assisted gas separation using the radial configurated cell with a flat membrane. The key requirement put forward for the membrane is high permeance; meanwhile, high selectivity is provided by the liquid absorbent. As was shown previously, the NH^3^/H_2_ selectivity gained up to 376 using the combined PVTMS-NH4SCN:EG system (the ideal membrane selectivity is 4.5), wherein the PVTMS permeance for pure ammonia is 3300 GPU.

The current study deals with a novel membrane-assisted gas separation cell designed for the hollow fibers. Unfortunately, there is no PVTMS hollow fibers production in the world, so it is of great importance to find the membrane with the mass transfer characteristics close to the mentioned ones. Moreover, there are very few papers providing data on ammonia permeance, especially for hollow fibers. Because of that, and in order to accurately determine the suitable membrane, the experimental study of PSF and PEI+PI membrane mass transfer properties was performed and the results are given in Table 4.

As is seen from Table 4, the PSF membrane provides quite high NH_3_ permeance, up to 1691 GPU; meanwhile, the PEI+PI membrane provides more than four times lower permeance. With respect to selectivity, both the PSF and PEI+PI membranes provide low selectivity for gas pair NH_3_/H_2_ of 3, wherein regarding NH_3_/N_2_, NH_3_/Ar, and NH_3_/CH_4_, the PEI+PI membrane is characterized by higher selectivity of 250.6, 148.5, and 143.2, respectively, comparing to PSF (77.9, 54, 73.5 for the same gas pairs). Despite the high selectivity for those gas pairs, both membrane materials could not provide sufficient separation for the NH_3_/H_2_ pair due to very low selectivity. Taking into account the composition of gas mixture to be separated (hydrogen is the matrix −62.53 mol% and ammonia is impurity −4.5 mol%), there is no sufficient ammonia partial pressure difference in the common membrane gas separation process to achieve the goal of the process—preferential capture of ammonia using the single stage. Because of that, and as the liquid absorbent provides the process’s selectivity, it is preferential to choose the more permeable membrane, so the polysulfone hollow fiber is the best choice for the membrane-assisted gas separation cell.

### 3.2. Membrane-Assisted Gas Separation Cell Performance: Helium Sweep Mode

**Figure 4:**
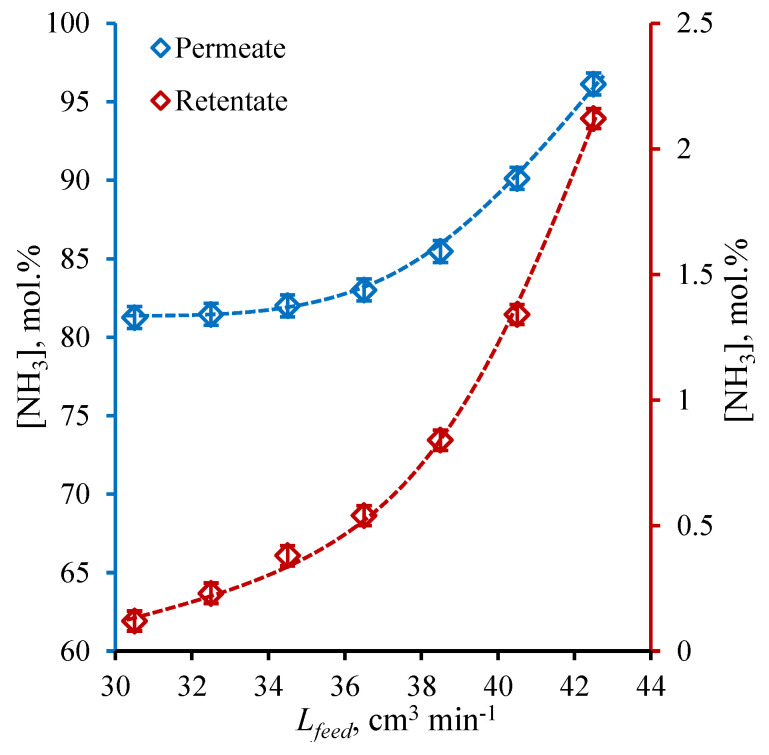
The NH_3_ concentration (mol%) in permeate and retentate flows under a helium sweep of cell’s bore as a function of feed flow rate (cm^3^ min^−1^) during a steady-state ammonia capture process.

**Figure 5:**
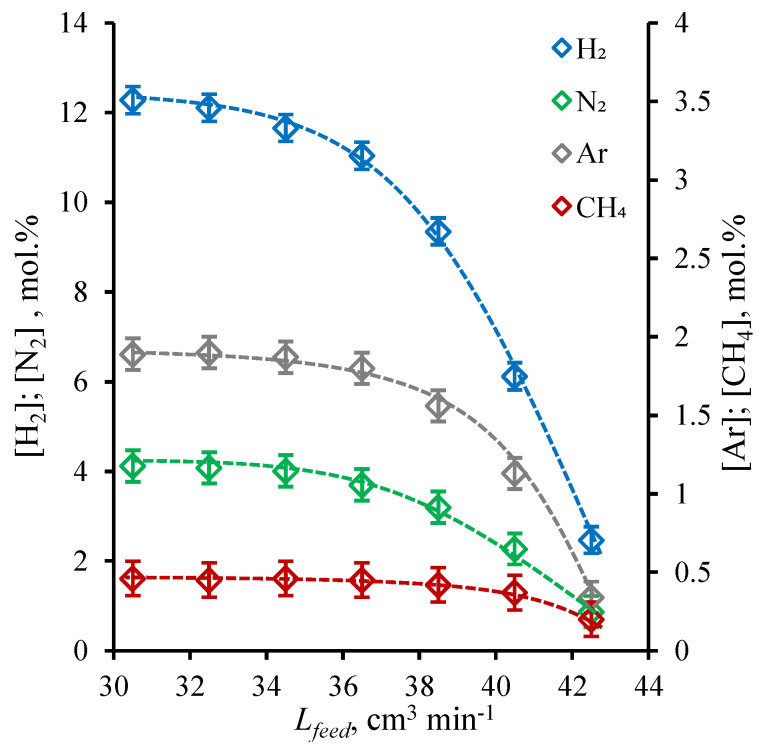
The H_2_, N_2_, Ar, and CH_4_ concentration (mol%) in permeate flow under a helium sweep of cell’s bore as a function of feed flow rate (cm^3^ min^−1^) during a steady-state ammonia capture process.

At first, the ammonia capture performance was tested under the sweep of the cell permeate side with a constant helium flow in order to evaluate the efficiency in ideal conditions. The results obtained during these ideal conditions performance test is given in Figure 4 and Figure 5, where the NH_3_ concentration values in permeate and retentate flows are shown as a function of feed flow rate (*L_feed_*, cm^3^ min^−1^) and the other components of the mix (H_2_, N_2_, Ar, and CH_4_) are plotted against the same value. As is seen from the graphs given in Figure 4, the NH_3_ content in the permeate stream rises over the whole range of considered feed flow rate, wherein the quite modest change in concentration is observed in the range of 30.5–36.5 cm^3^ min^−1^; meanwhile, the dramatic increase in NH_3_ content occurs in the range of feed flow rate of 38.5–42.5 cm^3^ min^−1^. The lowest ammonia content (81.3 mol%) in the permeate is obtained for the feed flow rate of 30.5 cm^3^ min^−1^ and the ultimate capture efficiency is achieved at 42.5 cm^3^ min^−1^ of feed flow −96.1 mol% of ammonia on the permeate side.

Considering the change in the ammonia content in the retentate flow, it is seen that the character of the graph tends to the linear dependency of feed flow rate in the whole range. It is seen that the increase in feed flow rate increases the ammonia concentration in the retentate flow and it reaches its maximum at the 42.5 cm^3^ min^−1^ of 2.12 mol%; meanwhile, the lowest value of 0.12 mol% is achieved at the lowest feed flow rate of 30.5 cm^3^ min^−1^. So, it is seen that the highest NH_3_ content in the permeate corresponds to the highest NH_3_ content in the retentate and opposite. In other words, there is a trade-off between the purity of ammonia to be captured and its recovery rate, which is typical for separation processes.

The obtained dependencies are well-explained by the features of the present separation process—membrane-assisted gas absorption. The liquid absorbent layer between two fibers is a virtually impermeable barrier for gases, which is characterized with low solubility in NH_4_SCN:Urea DES, and the mass transfer may only be provided by the diffusion of gas molecules through three barriers, namely two membranes and the liquid layer. The hydrogen molecule has the smallest kinetic diameter (except the ammonia) among other components of the mix [45,46], so the combined membrane-absorbent system allows the hydrogen to permeate in a very small amount, considering that the hydrogen partial pressure is the highest. At a low feed flow rate, the amount of ammonia is not sufficient to saturate all available absorbent volume, so other gases permeate too (Figure 5). Increasing the feed flow rate, the amount of ammonia, which is in contact with the combined membrane-absorbent system at the unit of time, is increased, so the continuous process of absorption–desorption on the opposite sides of the combined system generates growth in the ammonia content available for transfer across that system. At high feed fluxes, the combined system predominantly provides the NH_3_ ammonia mass transfer, so the other mix components remain at the upper side of the system and are removed with retentate. This enrichment of permeate with NH_3_ is accompanied by losses; as the greater amount of ammonia is supplied with increase in feed flow, the greater amount of it is not absorbed during contact, so the flow at the shell is being depleted of ammonia inefficiently and the retentate contains quite a lot of ammonia.

### 3.3. Membrane-Assisted Gas Separation Cell Performance: H_2_/N_2_ Mix Sweep Mode

**Figure 6:**
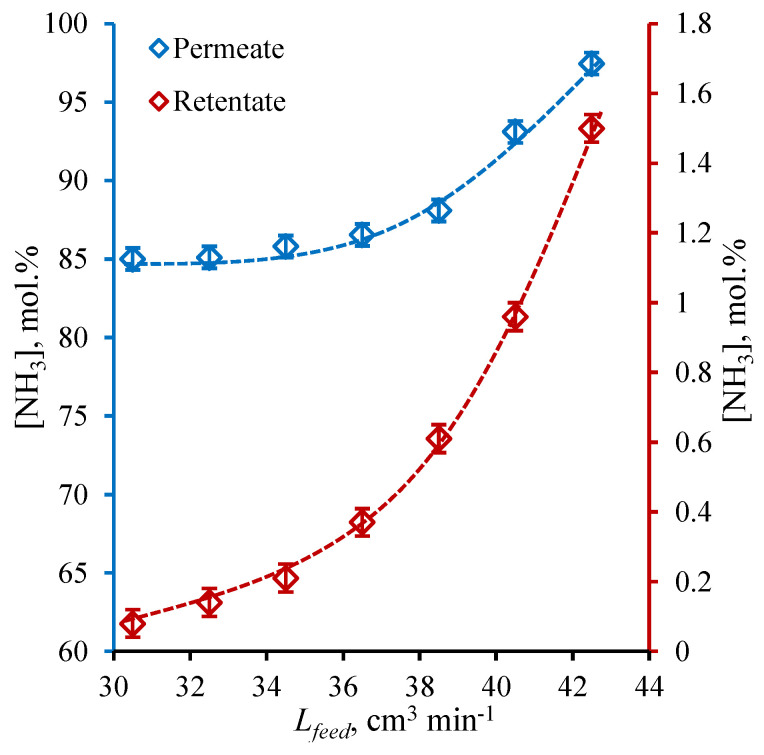
The NH_3_ concentration (mol%) in permeate and retentate flows under H_2_/N_2_ mix sweep of cell’s bore as a function of feed flow rate (cm^3^ min^−1^) during a steady-state ammonia capture process.

**Figure 7:**
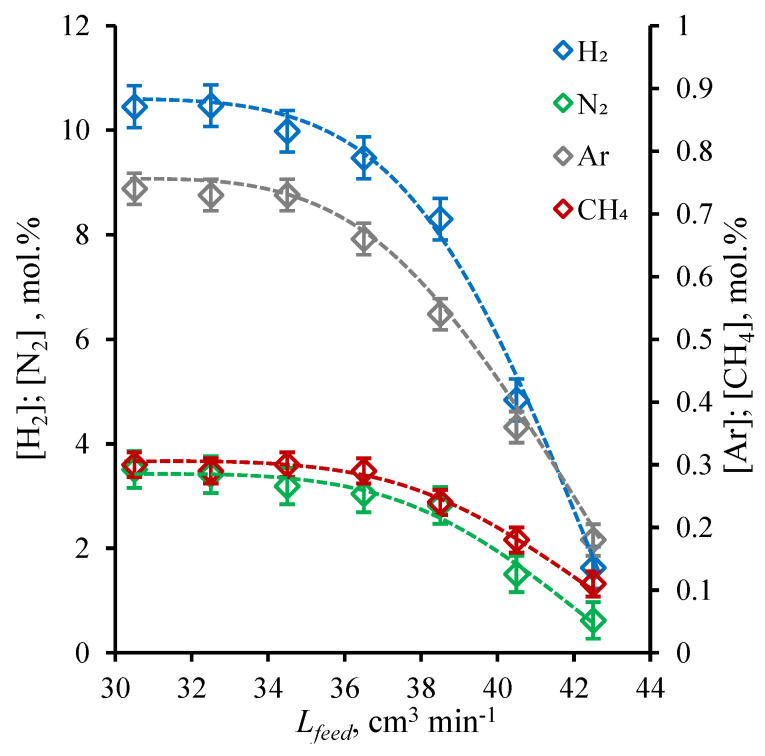
The H_2_, N_2_, Ar, and CH_4_ concentration (mol%) in permeate flow under H_2_/N_2_ mix sweep of cell’s bore as a function of feed flow rate (cm^3^ min^−1^) during a steady-state ammonia capture process.

Following the ideal conditions performance test, the membrane-assisted gas absorption cell was tested under the sweep of the permeate side with a constant H_2_/N_2_ mix in which a constant flow equals the previous one (30 cm^3^ min^−1^). The results obtained for these runs are represented as the graphs given in Figure 6 and Figure 7; the first shows the ammonia content in permeate and retentate plotted versus the feed flow rate, and the second shows the change in H_2_, N_2_, Ar, and CH_4_ concentrations over the observed feed flow rate range. In order to be able to compare the performance of the cell under these two experimental modes, in that case, the permeate flow content was determined taking into account the composition of sweep; in other words, the composition of the sum flow was determined and then, knowing the flow rate and its composition, the neat permeate flow composition was found.

As is seen from the graphs given in Figure 6, the characters of the obtained dependencies are similar to the previous one, and the NH_3_ concentration increases with an increase of feed flow rate over the whole observed range (30.5–42.5 cm^3^ min^−1^). Nevertheless, the NH_3_ concentration in permeate is slightly higher, especially in the range of feed flow rate from 30.5 to 36.5 cm^3^ min^−1^ (the growth of about 3.5–3.7 mol%). Increasing the feed, the growth in NH_3_ concentration slows down and the difference becomes lower than 3 mol%, and at the feed flow rate of 42.5 cm^3^ min^−1^, it is equal to 1.3 mol%. At this point, the ammonia concentration is 97.5 mol%, and at the feed flow rate of 30.5 cm^3^ min^−1^, it is equal to 85 mol%. Wherein, the NH_3_ content in retentate becomes lower when comparing the *helium sweep mode* and at the lowest observed feed flow rate, it is lower, 1 mol%, and the maximum observed NH_3_ concentration in the retentate is 1.5 mol%, which is lower by 0.6 mol%. As the character and the reason of these dependencies were discussed earlier, it is of great importance to explain the rise in the separation performance under the *H_2_/N_2_ mix sweep mode*. Here, the sweep contains the 75 mol% of hydrogen and 25 mol% of nitrogen; meanwhile, the feed contains the 62.5 mol% of hydrogen and 23.1 mol% of nitrogen and is supplied under the pressure of 0.4 MPa, so the partial pressure gradient is about 0.15 MPa in the case of H_2_ and 0.06 MPa in the case of N_2_, which is obviously lower than in the case of *helium sweep mode*; meanwhile, the ammonia partial pressure difference across the combined system remains unchanged. Because of that, the permeate is preferentially enriched with NH_3_ and is less enriched with H_2_ and N_2_, comparing the *helium sweep mode*.

Comparing the results obtained on the H_2_, N_2_, Ar, and CH_4_ content in the permeate (Figure 7) with the same values of *helium sweep mode*, it is seen that not only the H_2_ and N_2_ content decreased (affected by partial pressure difference drop), but Ar and CH_4_ concentration values became lower. Nevertheless, among these components, the H_2_ and N_2_ occupy the main place, and those concentrations change from 10.5 to 1.63 mol% and from 3.51 to 0.62 mol% for H_2_ and N_2_, respectively, increasing the feed flow rate from 30.5 cm^3^ min^−1^ to 42.5 cm^3^ min^−1^. These values are lowered by 0.84–1.83 and 0.25–0.82 mol% in the case of H_2_ and N_2_, respectively. Considering the Ar and CH_4_ content in the permeate flow, these values are lower, 0.8 mol% over the whole range of observed feed flow rate; meanwhile, under the *helium sweep mode*, they were up to 1.9 mol%.

### 3.4. The Steady-State Establishing Dynamic

**Figure 8:**
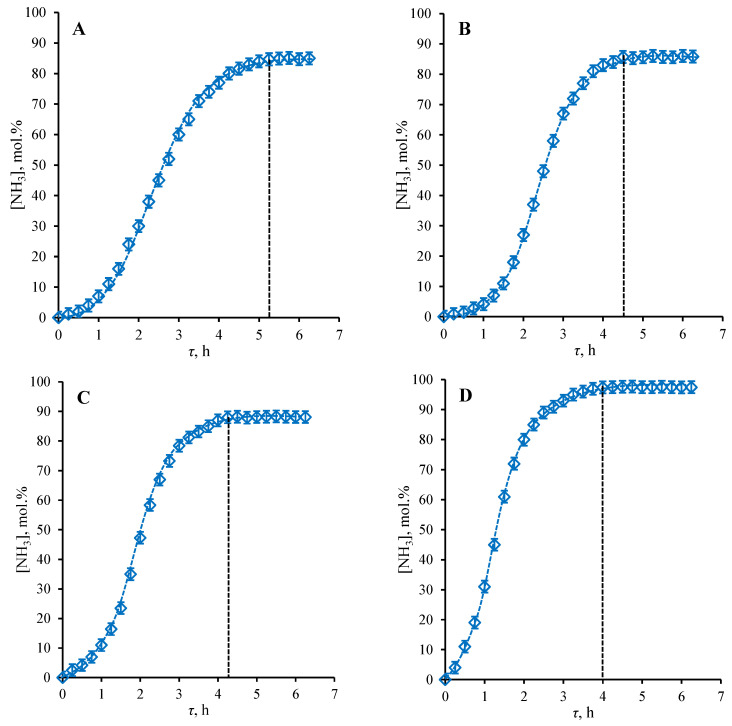
The NH_3_ concentration (mol%) in a permeate flow as a function of process operational time (h) during the establishment of the steady-state at 4 different feed flow rates (cm^3^ min^−1^): (**A**)—30.5 cm^3^ min^−1^; (**B**)—34.5 cm^3^ min^−1^; (**C**)—38.5 cm^3^ min^−1^; (**D**)—42.5 cm^3^ min^−1^.

The data obtained for the dynamics for establishing the steady-state mode of the separation process using the hollow-fiber-based membrane-assisted gas absorption cell are given in Figure 8 and represent the NH_3_ content in the permeate stream versus process duration under the *H_2_/N_2_ mix sweep mode* for four different feed flow rates (30.5, 34.5, 38.5, and 42.5 cm^3^ min^−1^). The reason to choose mix sweep mode is that this regime is close to the industry one, where authors propose to apply the present technique. Here, the steady-state was considered as having been achieved after the ammonia concentration value remained constant or the graph, which represents its concentrations, changed over the process duration time to form a plateau. The time required to establish the steady-state is determined as the first point of the graph, after which there is no change in NH_3_ content in the permeate. The time difference between each point is the GC analysis duration and it equals fifteen minutes.

As is seen from the graphs given in Figure 8, the time required to establish the steady-state is in the range of 4–5.25 h and it is a strong function of feed flow rate. Moreover, the time required to establish the steady-state decreases with an increase in feed flow rate; namely, in the case of feed flow rate of 30.5 cm^3^ min^−1^, the steady-state established 5 h and 15 min after the process was launched, increasing the feed flow rate up to 34.5 cm^3^ min^−1^ decreases the time to reach steady-state by 45 min, and the following growth in feed flow rate up to 38.5 and 42.5 cm^3^ min^−1^ reduces the required time by 15 min between each case.

As was mentioned previously, the increase in feed flow rate increases the amount of ammonia, which is in contact with the absorbent liquid layer at the unit of time. As the steady-state is established when the absorbent is completely saturated with ammonia, the higher feed flow rate provides faster reaching of the steady-state mode of the process. The DES used in this research was characterized with quite high ammonia absorption capacity of 146.3 g (NH_3_) per kg (DES) [12], so it required a few hours to establish the steady-state. In a recent study [7], it was found that for the same absorbent using the radial flat-sheet cell configuration, where the membrane area/absorbent volume ratio is much higher than the present case, the time required to establish the steady-state was equal to 3 h and 45 min. Despite the lowering of the amount of the absorbent, which means there is a shorter path for gas molecules to pass and lower volume of absorbent to saturate, the increase in the time to reach the steady-state compared to previous study is explained by the lower ammonia content in the feed; meanwhile, the recent study deals with a mix containing 15.5% of ammonia, the present one deals with 4.5 mol% of NH_3_ in the feed in the presence of four other components, the diffusion of which through the absorbent apparently affects the saturation of the DES.

## 4. Conclusions

Being a continuation of a membrane-assisted gas absorption technique study, the present study improves the efficiency of a separation cell in mass transfer through the optimization of unit configuration, decreasing the absorbent volume to membrane area ratio and implementing a combined system, which includes two types of hollow fibers. The examples covered in the present study include two different process modes: sweeping the permeate (bore) side of the cell with helium and with hydrogen-nitrogen mix, it was shown that the designed unit demonstrates high efficiency in the ammonia capture application in a single step.

As a result of the experimental study of the novel membrane-assisted gas absorption cell in ammonia capture application, it was shown that the ultimate achievable NH_3_ concentration in the permeate is 97.5 mol% (in the case of H_2_/N_2_ mix sweep mode); meanwhile, sweeping the bore side with helium provides the 96.12 mol% of ammonia in the permeate. Nevertheless, there is a trade-off between the product concentration and recovery rate, so the ultimate NH_3_ content in permeate is coupled with its losses in the retentate (up to 1.5 and 2.12 mol% residual NH_3_ under the H_2_/N_2_ and helium sweep mode, respectively).

## Figures and Tables

**Table 1:** Comparison of different NH_3_ absorbents.

Solvents	Mol W g mol^−1^	Density g sm^−3^	Viscosity, mPa s	Pressure kPa	T, K	Solubility NH_3_ g g^−1^	Refs
Choline-Chloride-based DESs							
ChCl + Urea (2:3)	91.89			10.8	313.2	0.002623	[18]
				104.8		0.024455	
				297.9		0.065991	
ChCl + Urea (1:2)	86.59			17.2	298.2	0.007561	
				95		0.037687	
				296.6		0.117149	
				11.3	313.2	0.003372	
				108.2		0.027231	
				302.1		0.073927	
ChCl + Urea (2:5)	82.8			19.8	313.2	0.004785	
				98.1		0.023076	
				299.1		0.068597	
ChCl + PhOH + EG (1:5:4)	142.92	1.09	29	101.3	298.2	0.16391	[19]
		1.085	12.5		313.2	0.11908	
ChCl + PhOH + EG (1:7:4)	153.8	1.08	12	101.3	313.2	0.13039	
ChCl + EG (1:2)	87.9			100.5	313.15	0.046	[20]
ChCl + Gly (1:2)	107.96			132.3		0.051	
ChCl + MU (1:2)	95.93			147.2		0.032	
ChCl + TA (1:2)	121.9			137.4		0.045	
ChCl + 1,4-BD (1:4)	100.02	1.0403	54.75	23.2	303.15	0.01276	[21]
				115.8		0.03408	
				396.9		0.15225	
		1.0348	36.66	33.3	313.15	0.0118	
				121.8		0.02498	
				389.2		0.11117	
ChCl + 1,4-BD (1:3)	102.5	1.0471	60.64	31.2	303.15	0.0163	
				113.7		0.04961	
				396.9		0.15075	
		1.0416	40.55	47.2	313.15	0.01557	
				131.7		0.04	
				416.6		0.10763	
ChCl + 2,3-BD (1:4)	100.02	1.0307	71.79	37.6	303.15	0.01335	
				120.9		0.04382	
				395.8		0.13483	
		1.0239	40.72	37	313.15	0.00908	
				124.1		0.03241	
				384		0.09424	
ChCl + 2,3-BD (1:3)	102.5	1.039	84.88	32.4	303.15	0.01337	
				121		0.04596	
				384.8		0.12796	
		1.0325	48.73	49	313.15	0.01402	
				130.4		0.0353	
				390.4		0.0978	
ChCl + 1,3-PD (1:4)	88.8	1.0705	34.45	32.7	303.15	0.02166	
				132.8		0.06788	
				391.5		0.16814	
		1.0648	24.21	31.6	313.15	0.01197	
				121.8		0.04286	
				390.7		0.11846	
ChCl + 1,3-PD (1:3)	91.98	1.0753	40.05	34	303.15	0.01448	
				131.7		0.05489	
				396.2		0.14728	
		1.0697	27.84	31.4	313.15	0.01083	
				128.5		0.0428	
				401.4		0.11376	
ChCl + ImZ + EG (3:7:14)	73.52	1.105	15	11	313.2	0.00848	[22]
				101.6		0.0836	
ChCl + TrZ + EG (3:7:14)	73.79	1.126	15	9.1		0.02176	
				104.3		0.11061	
ChCl + TetrZ + EG (3:7:14)	74.1	1.158	12	7.7		0.07313	
				104.9		0.16948	
ChCl + Res (1:3)	117.48			101.3	313.2	0.053	[23]
ChCl + Phe (1:3)	105.49					0.081	
ChCl + EG (1:2)	87.9					0.041	
ChCl + PhA (1:2)	137.31					0.043	
ChCl + Gly (1:2)	107.96					0.053	
ChCl + Phe + EG (1:3:5)	81.367					0.091	
ChCl + Phe + Gly (1:3:5)	98.046					0.095	
ChCl + Res + Gly (1:3:5)	103.375			101.3	293.15	0.18	
					298.15	0.17009	
		1.21			313.15	0.1303	
ChCl + D-fructose + Gly (1:3:5)	126.73			101.3	313.15	0.11027	
ChCl + DL-malic Acid + Gly (1:3:5)	111.37					0.045	
ChCl + Levulinic Acid + Gly (1:3:5)	105.38					0.055	
ChCl + Oxalic Acid + Gly (1:3:5)	96.69					0.074	
ChCl + Malonic Acid + Gly (1:3:5)	101.36					0.081	
ChCl + Phenylacetic Acid + Gly (1:3:5)	112.06					0.097	
Ethylamine-hydrochloride-based DESs							
EaCl + Urea (2:1)	74.38	1.103	97.8	8.5	313.2	0.00882	[24]
				98.5		0.07486	
				301.7		0.1877	
EaCl + Urea (1:1)	70.81	1.142	197.7	9.5		0.01102	
				99.1		0.07788	
				300.6		0.179	
EaCl + Urea (1:2)	67.23	1.179	105.5	8.4		0.00897	
				96.3		0.07117	
				296.2		0.17134	
EaCh + Gly (1:2)	88.6			101.3	313.15	0.114	[25]
EaCl + Gly (1:5)	90.3			101.3	313.2	0.129	[26]
EaCl + Res + Gly (1:1:5)	93.15					0.149	
EaCl + Res + Gly (1:2:5)	95.3					0.163	
EaCl + Res + Gly (1:3:5)	96.9					0.174	
EaCl + Res + Gly (1:4:5)	98.2	1.244				0.181	
EaCh + PhOH (1:2)	166.0			101.3	313.15	0.119	[27]
EaCl + PhOH (1:5)	187.1				313.2	0.138	
EaCl + PhOh (1:7)	192.4				298.2	0.167	
Ammonium-thiocyanate-based DESs							
NH_4_SCN + Gly (2:3)	85.7	1.239	71.18	101.3	313.2	0.17642	[12]
NH_4_SCN + EG (1:3)	65.58	1.138	11.46			0.1684	
NH_4_SCN + Urea (2:3)	91.3	1.256	41.04			0.1463	
NH_4_SCN + AT (2:3)	65.89					0.0918	
NH_4_SCN + CL (2:3)	98.34					0.0295	
NH_4_SCN + Im (1:2)	70.76	1.115	17.47	100	303.15	0.164	[28]
		1.106	13	100	313.15	0.117	
NH_4_SCN + Im (1:3)	70.09	1.125	18.22	100	303.15	0.122	
Other DESs							
MAA + TetrZ (2:1)	72.08	1.05	33	9	313.2	0.07834	[29]
				102.9		0.13624	
Tri + Gly (1:3)	86.32	1.249	163	101.3	303.15	0.147	[30]
Im + Gly (1:3)	86.09					0.126	
Tz + Gly (1:3)	86.58					0.179	
[bmim][MeSO_3_] + Urea (1:1)	147.19	1.189	295.72	172.6	313.2	0.01787	[31]
[Im][NO_3_] + EG (1:3)		1.213	9.8	101.3	313.15	0.172	[32]
[Mim][NO_3_] + EG (1:3)		1.186	8.7			0.152	
[Mmim][NO_3_] + EG (1:3)		1.175	9			0.141	
Im + Res (1:1)	89.09	1.2017		101.3	313.15	0.154	[33]
Im + Res (1.5:1)	84.89			101.3	313.15	0.128	
Im + Res (2:1)	82.08			101.3	313.15	0.107	
Im + Res (2.5:1)	80.08			101.3	313.15	0.101	
[Me_2_C^OH^_2_N]Cl + U (1:1)	114.86	1.211	723.38	101.3	313.2	0.035	[13]
[MeC^OH^_3_N]Cl + U (1:1)	129.96	1.250	1026.3			0.045	
Ionic Liquids							
[mim][Tf_2_N]	362.24			6.496	313	0.04983	[34]
				102.71		0.12599	
				610.14		0.27785	
[eim][Tf_2_N]	376.27	1.559711	33.165	8.179		0.04979	
				105.12		0.12356	
				622.99		0.28106	
[mmim][Tf_2_N]	376.27	1.569724	52.96	14.488		0.043	
				98.631		0.10862	
				610.92		0.25934	
[bim][SCN]	182.26	1.084584	152.28	51.718	303	0.16445	
				98.817		0.20369	
				546.09		0.51297	
		1.078656	84.302	46.817	313	0.14483	
				96.588		0.18314	
				563.18		0.42981	
[bim][NO_3_]	186.18	1.172231	248	100.09	303	0.13721	
				553.89		0.43814	
		1.165499	136.95	86.01	313	0.10611	
				141.53		0.15733	
				515.83		0.34301	
[bmim][SCN]	197.3	1.068149	45.263	72.441	303	0.01381	
				144.51		0.02762	
				559.47		0.13897	
		1.062258	30.461	82.918	313	0.01295	
				203.46		0.03107	
				556.94		0.09667	
[bmim][DCA]	223.3	1.058303	25.093	61.162	303	0.0122	
				114.28		0.02288	
				567.88		0.15329	
		1.05196	18.133	62.376	313	0.00763	
				128.32		0.01678	
				565.56		0.1022	
[bmmim][Tf_2_N]	433.39	1.414188	77.731	50.322	303	0.00393	
				119.43		0.00982	
				536.8		0.06287	
		1.404927	49.703	100.49	313	0.00786	
				216.34		0.01729	
				607.72		0.06012	
[bmmim][SCN]	211.33	1.068578	192.26	95.668	303	0.01451	
				184.62		0.0282	
				544		0.10395	
		1.062887	108.5	40.706	313	0.00484	
				149.89		0.01692	
				560.58		0.07656	
[bmmim][DCA]	237.33	1.053082	47.66	115.38	303	0.01794	
				199.86		0.03157	
				560.58		0.11553	
		1.04702	31.616	103.42	313	0.00861	
				174.97		0.02081	
				569.34		0.08252	
[bmim][BF_4_]	226.02	1.203	123.41	101.3	298.2	0.01701	[35]
				130		0.01543	
				200	323	0.01027	
				220	298.15	0.03505	[36]
				630		0.14856	
				80	313.15	0.00827	
				610		0.09648	
				500	293	0.07535	[37]
[bmim][PF_6_]	284.18			101.3	298.2	0.02101	[35]
				170		0.03227	
				270	323	0.02448	
[bmim][Tf_2_N]	419.36		60.7	101.3	299.4	0.0053	
[emim][Tf_2_N]	391.31			140	323	0.0065	
				170		0.0043	
[hmim][Cl]	202.72			100	323	0.00536	
				130		0.02509	
[emim][BF_4_]				101.3	333.15	0.0095	[38]
[emim][Tf_2_N]	419.36					0.0065	
[emim][NO_3_]						0.0097	
[EtOHmim][SCN]		1.2	55		313.15	0.031	
[EtOHmim][NO_3_]		1.32	90			0.02	
[EtOHmim][PF_6_]		1.54	92			0.038	
[EtOHmim][BF_4_]	213.97	1.35	54.09			0.04502	
[EtOHmim][Tf_2_N]		1.57	40			0.036	
[EtOHmim][DCA]	193.21	1.18	38.83			0.018	
[emim][Ac]	170.11			101.3	298.3	0.03202	[39]
				590	298	0.14406	
				540	323	0.11752	
[emim][SCN]	169.24			101.3	298.1	0.04502	
				440	298	0.07906	
				420	323	0.07287	
[emim][EtOSO_3_]	236.29			520	298	0.07808	
				480	323	0.06653	
				470	323	0.10123	
[DMEA][AC]	149.19			101.3	298.1	0.10006	
				470		0.10123	
[emim][BF_4_]	197.97			110	298.15	0.01487	[36]
				550		0.13893	
				140	313.15	0.01235	
				620		0.09535	
[hmim][BF_4_]	254.08			220	298.15	0.03891	
				600		0.15447	
				230	313.15	0.02507	
				600		0.09139	
[omim][BF_4_]	282.17			120	298.15	0.02333	
				610		0.17877	
				180	313.15	0.02385	
				600		0.10908	
[TMGH][BF_4_]	201.98			101.3	293.2	0.09006	[37]
				520	293	0.09134	
[TMGH][NTf_2_]	395.32			540	293	0.05057	
[TMGHPO_2_][BF_4_]	264.95			101.3	293.2	0.038	
				420	293	0.04654	
[MTEOA][MeOSO_3_]	275.32	-	440.01		313	0.082	[40]
IL-4		1.184	5389.8	101.3	313.15	0.012	[17]
IL-5		1.218	3366.5			0.028	
[HOOC]_17_-COFs				100	298	0.15915	[41]
[HOOC]_33_-COFs				100	298	0.1399	
[HOOC]_0_-COFs				100	283	0.15728	
[HOOC]_0_-COFs				100	298	0.11672	
[HOOC(CH_2_)_3_mim][Tf_2_N]	449.23		1592		313	0.058	[42]

**Table 2:** GC analysis details used for membrane-assisted gas absorption cell separation performance test.

9	Characteristics
Detectors	TCD №1, 393.15K TCD №2, 463.15 K TCD №3, 463.15 K
Columns	15% PEG-600 on PTFE, 333.15 K 60/80 mesh 3 m × 2 mm i.d. stainless steel tube (TCD №1) 333.15 K CaA 5A, 333.15 K 60/80 mesh 2 m × 2 mm i.d. stainless steel tube (TCD №1) 333.15 K Hayesep B, 333.15 K 60/80 mesh 3 m × 2 mm i.d. stainless steel tube (TCD №2) 333.15 K Hayesep Q, 333.15 K 60/80 mesh 2 m × 2 mm i.d. stainless steel tube (TCD №3) 333.15 K
Sample loop	0.25 cm^3^, 383.15 K (TCD №1) 1 cm^3^, 453.15 K (TCD №2, 3)
Carrier gas	He ≥ 99.995 vol.% (TCD №1, 2, 3) 20 cm^3^ min^−1^

**Table 3:** Membrane-assisted gas absorption process experimental conditions.

Parameter	Value
Feed pressure	0.4 MPa
Permeate pressure	0.132–0.135 MPa
Temperature	298.15 K
Feed flow rate	30.5–42.5 cm^3^ min^−1^
Retentate flow rate	30 cm^3^ min^−1^
Helium sweep flow rate	30 cm^3^ min^−1^
H_2_/N_2_ mix sweep flow rate	30 cm^3^ min^−1^

**Table 4:** PSF and PEI+PI hollow fiber membrane permeance for pure gases.

	Permeance, GPU ^a^		α(NH_3_/*x*)	
	PSF	PEI + PI	PSF	PEI + PI
NH_3_	1691	400.9	-	-
H_2_	563	135.6	3	3
N_2_	21.7	1.6	77.9	250.6
Ar	31.3	2.7	54	148.5
CH_4_	23	2.8	73.5	143.2

@ 101.325 kPa transmembrane pressure and 298.15 K. ^a^ 1 GPU = 1 × 10^−6^ cm^3^ cm^−2^ s^−1^ cmHg^−1.^

## Data Availability

Not applicable.
